# Progression of the leprosy reaction and nerve damage: A prospective cohort study in children with leprosy from the Brazilian Amazon

**DOI:** 10.1371/journal.pntd.0012772

**Published:** 2024-12-19

**Authors:** Sabrina Sampaio Bandeira, Andressa Bocalon dos Anjos, Carla Avelar Pires, Juarez Antonio Simões Quaresma

**Affiliations:** 1 Sanitary Dermatology Referral Unit “Dr. Marcello Cândia”, Secretary of State for Public Health, State of Pará, Marituba, Brazil; 2 Postgraduate Program in Biology of Infectious and Parasitic Agents, Institute of Biological Sciences, Federal University of Para, Belem, PA, Brazil; 3 Tropical Medicine Center, Federal University of Para, Belem, PA, Brazil; 4 Center of Health and Biological Sciences, State University of Para, Belem, PA, Brazil; Adolfo Lutz Institute of Sao Jose do Rio Preto, BRAZIL

## Abstract

**Introduction:**

Leprosy reactions being closely related to damage to peripheral nerves and physical disabilities associated with disease progression. Disabilities in childhood can have a devastating effect with impaired children’s cognitive, emotional, social, and educational development. We followed up the progression of leprosy reactions in children identifying associated factors with the emergence and/or worsening of nerve damage.

**Materials and methods:**

A prospective longitudinal cohort study was performed in patients under 15 years of age affected by leprosy reactions diagnosed at the Leprosy referral unit in the Amazon region of Brazil. The follow-up time was a cohort for 2 years from diagnosis. A clinical evaluation and the simplified neurological were performed at diagnosis and every 6 months, until the end of the follow-up period.

**Principal findings:**

Of the 77 children monitored, 38 had reactions and were recruited. Only 23.7% (9/38) of patients improved their initial neurological impairment and 42.1% (16/38) had progression of the leprosy reaction with worsening of the initial nerve damage. Two or more reaction episodes, nerve damage and two or more nerves affected at diagnosis, isolated neuritis, silent neuritis and low educational level of caregiver had a significant association with the emergence and/or worsening of neural damage.

**Conclusions:**

A high proportion of children had progression of the leprosy reaction with worsening neural damage. The absence of warning signs of inflammation, such as pain and exacerbation of skin lesions, appears to contribute to the worse prognosis of reactions. Early diagnosis, systematic monitoring especially of neural function, beyond the educate caregivers to recognize the reactional episode early and to helping with children’s self-care are very important measures to minimize the emergence or worsening of neural damage resulting from leprosy reactions.

## Author summary

Reactions are complications of leprosy which lead to acute inflammation of the skin and/or nerves. When these inflammations occur in peripheral nerves, they are the main cause of loss of sensitivity, muscle paralysis and permanent deformities. We accompany children who had leprosy reactions observing how evolve and which factors are associated with the worsening of reactions. To this end, we carried out a cohort study in patients under 15 years of age affected by leprosy reactions diagnosed at the Leprosy referral unit in the Amazon region of Brazil. The children were followed for 2 years since diagnosis, with clinical and strength and sensitivity of nerves assessments. Of the 38 children recruited, a large proportion (42.1%) had worsening nerve function. Furthermore children who had repeated episodes of reactions, nerve damage and multiple nerves affected at diagnosis, deterioration of neural function without pain and/or without exacerbation of the skin lesions, and with caregivers with a low level of education, showed worse development of the reaction. Measures for early diagnosis, constant monitoring of nerve functions and information about the disease for caregivers are important to minimize disabilities, especially in childhood.

## Introduction

Leprosy is a chronic infectious disease caused by *Mycobacterium leprae*, that affects mainly the skin and peripheral nerves. At any time during the chronic course of the disease: before, during or after multidrug therapy (MDT); especially in the most severe multibacillary clinical forms, a significant proportion of patients develop imune-mediated reactions. The inflammatory process arising from the leprosy reaction can result in neuritis with deterioration of autonomous, sensory and/or motor neural function, can lead to permanent nerve damage and visible physical deformities [1].

Although reactions are more common in adults, they are also present in children. [2]. For a long time, due to the long incubation period, leprosy in children was synonymous with the disease in its mildest clinical forms, paucibacillary [3,4], however in recent years several studies have shown a high proportion of children with multibacillary clinical forms and complications such as reactions have been increasingly frequent in pediatric age [5–10].

The Brazilian Amazon is a highly endemic region of disease where delays in diagnosing children, with the need for many doctors until the disease is confirmed are frequent situations, thus representing a region of great challenge for brazilian leprosy control programs [5,11]. Expected that in the next years, this situation will be even more aggravated due to the COVID-19 pandemic, when many children stayed at home being in contact with untreated cases, in addition to the interruption of health services and active searches. Thus, there will be a potential increase in the number of children diagnosed with the multibacillary forms may suffer reactions and therefore with high risk of developing physical disabilities [12].

The burden of a child with visible deformities resulting of a disease with millenial stigma such as leprosy, seems to be even greater than that of an adult, as it has long-term implications with impaired children’s cognitive, emotional, social, and educational development [13,14].

Faced with this critical scenario, this study was conducted to accompany a cohort of childrens with leprosy reactions diagnosed at the Health Referral Unit “Marcello Candia” (HRUMC) in Brazilian Amazon, observing how reactions evolve in this age group and which factors are associated with the worsening of reactions or neurological impairment. It is expected that the results will be used as input for the implementation measures to prevent the onset or worsening of physical disabilities in children with leprosy.

## Materials and methods

### Ethics statement

This study was approved by the Ethics Committee on Human Research of the Institute of Health Sciences, Federal University of Pará (Approval No. 1.059.013) and was conducted in accordance with the Declaration of Helsinki (1964) and its subsequent revisions. The anonymity of all participants was respected. An informed consent form was signed by the patient or legal representative as well as by children who were 12–14 years of age and agreed to participate in the study and the photo documentation publicity.

A prospective longitudinal study was performed in patients under 15 years of age affected by leprosy reactions at diagnosis, during or after leprosy treatment. These patients were diagnosed, treated and followed up at the HRUMC, leprosy reference center specialized, located in the municipality of Marituba, in the state of Pará, high leprosy endemic region in the Amazon, northern Brazil.

All leprosy patients under 15 years of age diagnosed from April 2014 to June 2015 and from January 2019 to March 2020 were invited to participate in the study (convenience sampling). The follow-up time was a cohort for 2 years from diagnosis. Thus, the study population consisted of patients who during this period had some leprosy reactional episode. Patients who lacked cognitive capacity or had difficulty understanding the instructions for the strength and sensory tests in the simple neurologic examination, who had a diagnosis of another associated neurologic disease, or who abandoned treatment and/or did not return for follow-up were excluded.

Patients were diagnosed by a committee of leprosy experts based on the cardinal signs of the disease and was supported by findings of histopathology and skin smear microscopy [15]. Leprosy reactions were classified into type 1, type 2 and isolated neuritis. The type 1 reaction was characterized by acute inflammation in skin lesions or nerves and the type 2 reaction as the inflammation cutaneous nodules with or without neuritis [16]. While isolated neuritis was considered when it was not associated with other clinical signs and symptoms of reactions [2]. Silent neuritis was defined as a change in neural function without neural pain, while the overt neuritis was characterized with peripheral nerve pain associated or not with deterioration of neural function [17]. To determine the number of reactions, exacerbation of the lesions and/or alteration of the neural function, seven days or more after completion of the reaction treatment was considered to be a new reaction and when these alterations appeared in less than seven days were considered to be a continuation of the previous reaction. All procedures carried out with patients, such as drug treatment for leprosy and reactional episodes, in addition to rehabilitation of disabilities were conducted according to the guidelines of the World Health Organization and Brazilian Ministry of Health [15].

At the time of diagnosis patients aged under 15 years and their guardians were interviewed using a structured questionnaire when clinical and epidemiological data were collected. A clinical evaluation and the simplified neurological were also performed at diagnosis, and patients were reassessed every 6 months, or at any sign or symptom of leprosy reaction until the end of the follow-up period.

The simplified neurological assessment was performed according to the standard procedures established by World Health Organization and the Brazilian Ministry of Health (2016). This exam involved inspection, nerve palpation, muscle strength testing, and sensory evaluation of the eyes and upper and lower limbs; the degree of physical disability was also assessed. Thus, sensibility testing was performed using standard set of 6 coloured Semmes-Weinstein monofilaments. However, a score was assigned per tested site ranging from 0 to 6. A score of 6 was given when the thinnest monofilament (0,05g) in the test series was felt; a score of 0 if even the thickest filamento (300g) was not felt. In addition, two new sensory evaluation points were added, one on the dorsum of the hand and the other on the dorsum of the foot, to assess the sensitivity of the radial and peroneal nerves, respectively. The motor damage was evaluated by Voluntary Muscle Testing (VMT) and graduation score was used for muscle weakness was from 0 = paralysis to 5 = normal muscle function. The grade of physical disability was classified according to the established standard: grade 0 (no leprosy-related physical disability), grade 1 (decreased strength and/or loss of protective sensitivity) and grade 2 (presence of visible disabilities). It was not possible to assess pain using the visual analogue scale due to the young age of some children who participated in the study, in addition to their low educational level. Thus, pain was evaluated only for the presence or absence of pain.

The progression of the leprosy reaction was defined as the onset or worsening of neurological impairment, while non-progression was the unchanged or improvement of the initial neurological condition.

A comparison was made between the simplified neurological assessments at the time of diagnosis and post-MDT period. Patients who had a reduction in the sum of strength and sensitivity scores and/or with the appearance of visible deformities, were considered to have worsened neurological impairment. While patients who had an increase in these values, without the appearance of visible deformities, were considered to have improved neural damage. In addition, patients who maintained the same values without the appearance of new deformities, were classified as unchanged in the neurological status.

Results were analyzed using GraphPad Prism version 5.0 software (GraphPad Software, Inc., San Diego, California). A descriptive analysis with calculation of the absolute and relative frequencies of the categorical variables was performed. To verify the degree of association between progression of the leprosy reaction and clinical and epidemiologic variables, we used the Fisher’s exact test because of our small sample size. Progression of the leprosy reaction was considered as the dependent variable. The considered independent variables were: gender (male and female); age group (0–9 and 10–14); time from diagnosis (up to one year and more than one year); caregivers (parents and others); caregiver’s educational level (incomplete elementary school and complete elementary school or more years of study); household income (up to one minimum wage and more than one minimum wage); clinical form (borderline and others); operational classification (paucibacillary and multibacillary); presence of nerve damage at diagnosis (yes/no), number of nerve damage at diagnosis (up to one and two or more); number of episodes of leprosy reaction (only one and two or more); leprosy reaction at diagnosis (yes/no); type of reaction (isolated neuritis and reaction with skin lesions) and silent neuritis (yes/no). A value of p ≤ 0.05 was considered significant.

## Results

There were 77 children diagnosed and followed up at the HRUMC during the period of study; of these, almost half 49.3% (38/77) had leprosy reactions.

The present study included 38 children who had leprosy reaction, 27 (71.1%) of whom were males boys and 24 (63.2%) patients were in the 10–14 year age group. The youngest participant was only three years old. The most, 21 (55.3%) of children had their parents as caregivers, and education level of caregivers had incomplete elementary school in 21 (55.3%) cases. A household income of up to 1 minimum wage was reported by 31 (81.6%) of the responsible persons interviewed. The time from symptom onset to diagnosis was more than one year in 22 (57.9%) patients. The borderline clinical form was predominant ocurred in 27 (71.1%) children and according to the operational classification 35 (92.1%) cases had multibacillary leprosy (Table 1).

**Table 1:** Clinical and epidemiologic characteristics of 38 study participants.

Characteristics	n	%
**Sex**		
Male	27	71.1
Female	11	28.9
**Age group (y)**		
0–9	14	36.8
10–14	24	63.2
**Caregivers**		
The parentes	21	55.3
Others	17	44.7
**Educational level of caregivers**		
Incomplete elementary school	21	55.3
≥ Complete elementary school	17	44.7
**Household income**		
≤1 Minimum wage	31	81.6
2–3 Minimum wages	7	18.4
**Time to diagnosis (y)**		
≤1	22	57.9
>1	16	42.1
**Clinical forms**		
Indeterminate	0	0
Tuberculoid	3	7.9
Borderline	27	71.1
Lepromatous	5	13.1
Primarily neural	3	7.9
**Operational classification**		
Paucibacillary	3	7.9
Multibacillary	35	92.1

**Fig 1:**
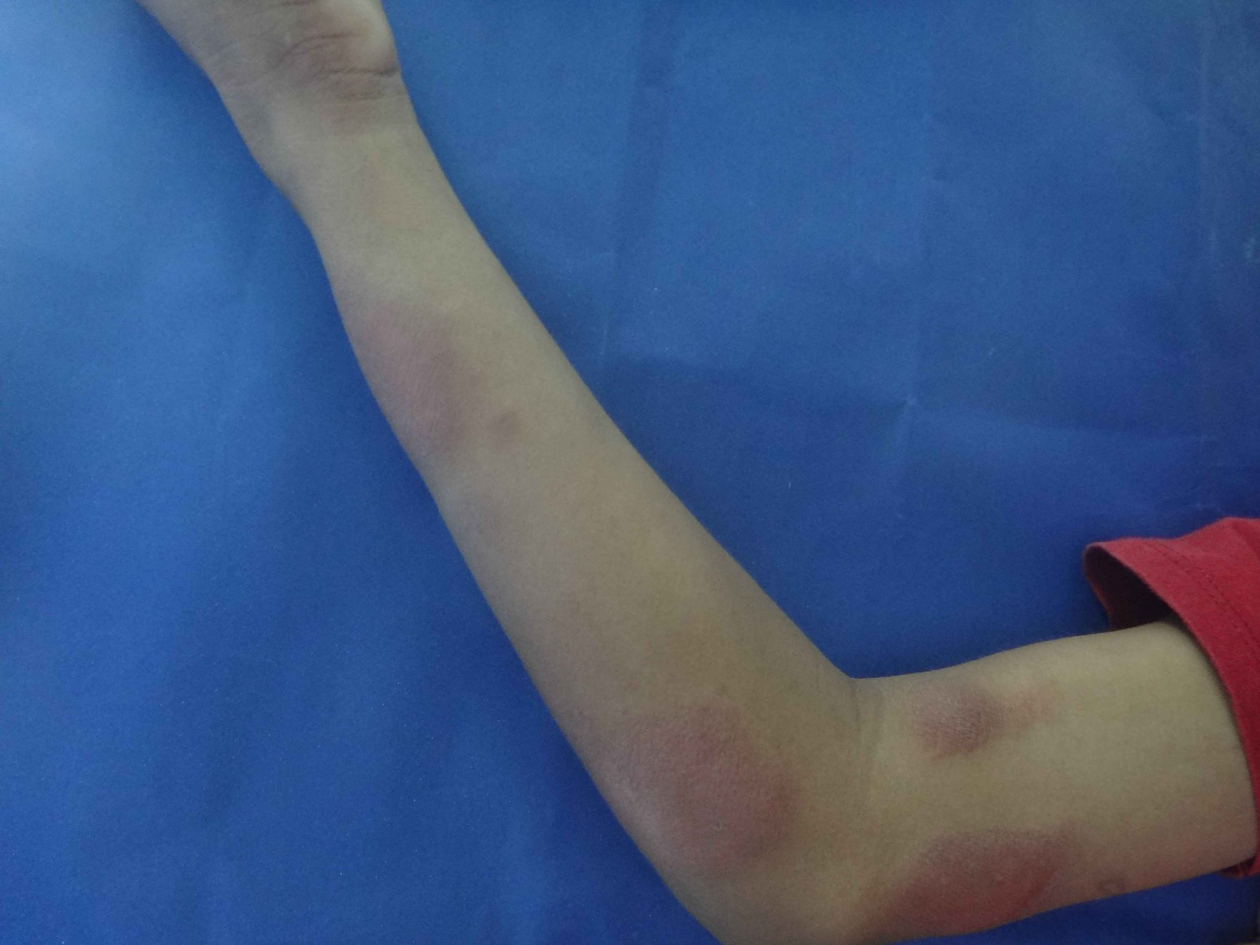
Type 1 reaction: right upper limb of the child with exacerbation/inflammation of skin lesions.

**Fig 2:**
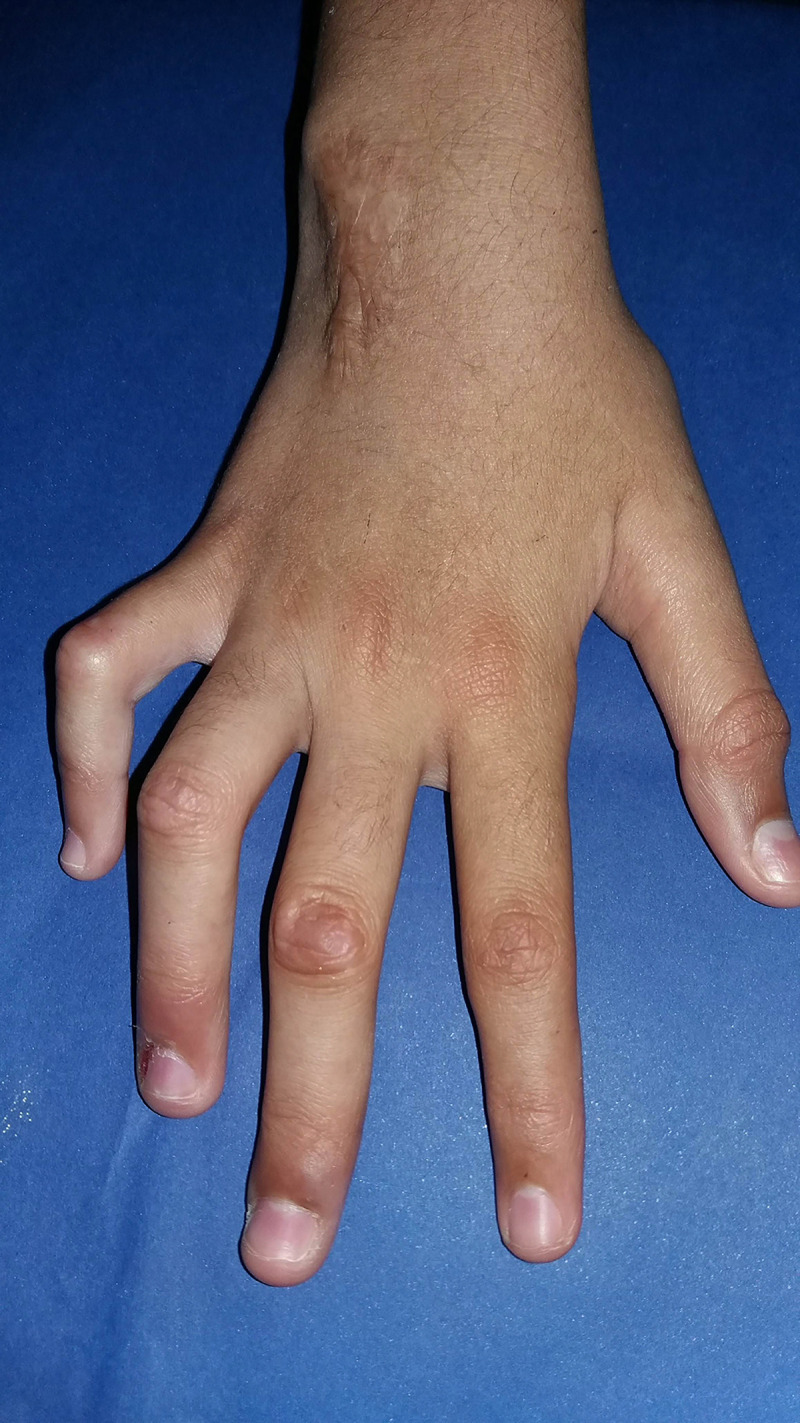
Median-cubital claw deformity right hand: two nerves affected at diagnosis.

**Fig 3:**
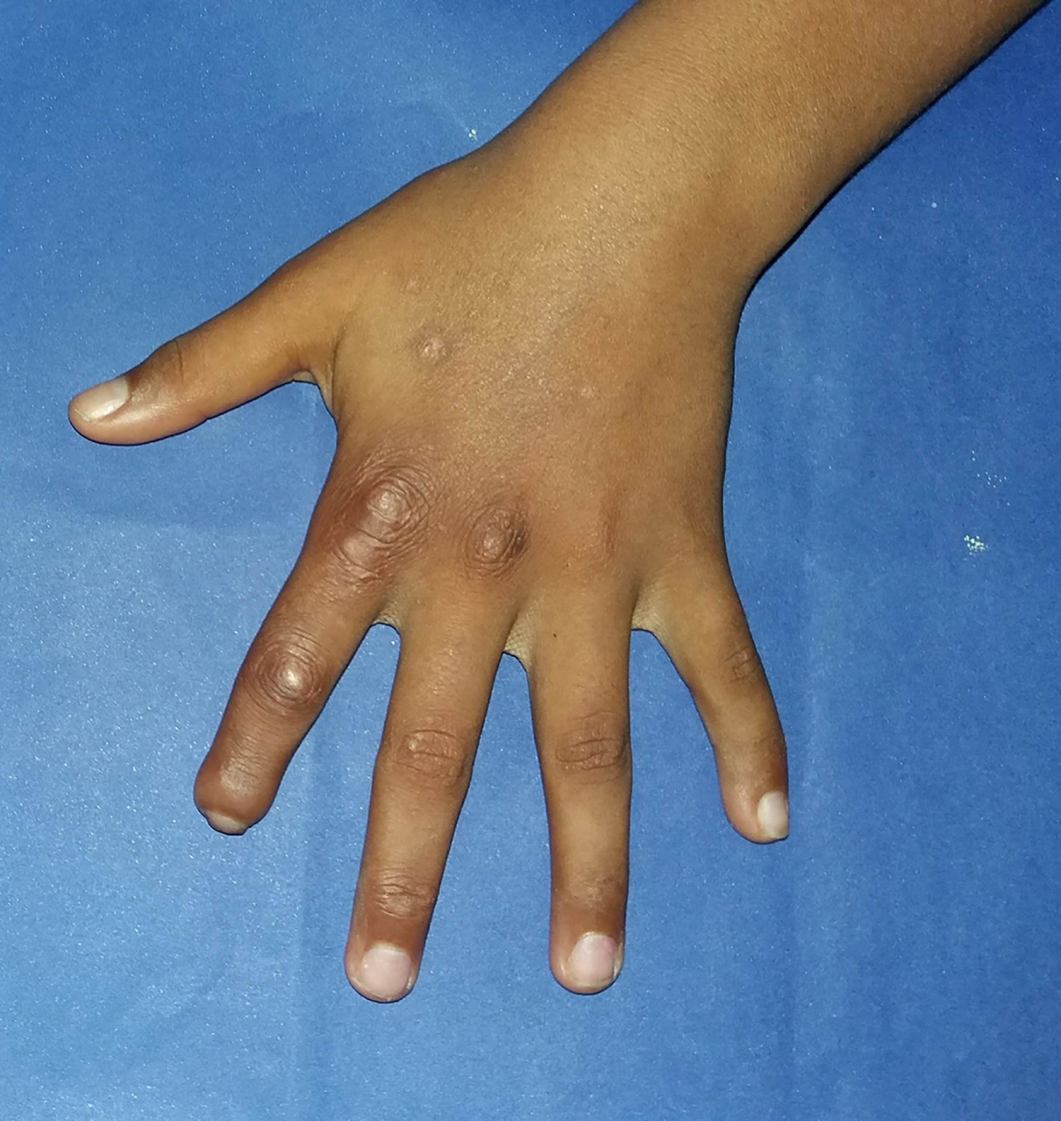
Absorption of bone deformity in 2th finger of the left hand at diagnosis: deformity that characterize degree two of physical disability.

The type 1 reaction was most frequent observed in 18 (47.4%) patients (Fig 1), followed by isolated neuritis occurred in 16 (42.1%) of cases. Twenty-eight (73%) of children had two or more reactional episodes and the mean was 2.3 episodes. The majority of patients 27 (71.1%) had reactions during MDT treatment, although, more than half of the participants 22 (57.9%) had reactions after the end of treatment. Nerve damage was observed in 29 (76.3%) participants, 23 (60.5%) have been diagnosed with peripheral nerve damage, and 12 (31.6%) patients had two or more nerves affected at the time of diagnosis (Fig 2). Most of the children in the study 20 (52.6%) had some physical disability already at diagnosis and 11 (28.9%) had grade 2 of disability (Fig 3). Regarding pain the silent neuritis occurred in 16 (42.1%) of patients (Table 2).

**Table 2:** Characteristics of the reactions and nerve damage of the study patients.

Variables	n	%
**Reaction types**		
Type I	18	47.4
Type II	4	10.5
Isolated neuritis	16	42.1
**Number of reaction episodes**		
Only one	10	26.3
2 or more	28	73.7
**Time reaction occured**		
At diagnosis	24	63.1
During treatment	27	71.1
After treatment	22	57.9
**Nerve damage**		
Yes	29	76.3
No	9	23.7
**Nerve damage at diagnosis**		
Yes	23	60.5
No	15	39.5
**Number of nerves affected at diagnosis**		
0	15	39.5
1	11	28.9
2 or more	12	31.6
**Neuritis**		
No neuritis	9	23.7
Silent	16	42.1
Overt	13	34.2
**Degree of physical disability at diagnosis**		
0	18	47.4
1 2	9 11	23.7 28.9

**Fig 4:**
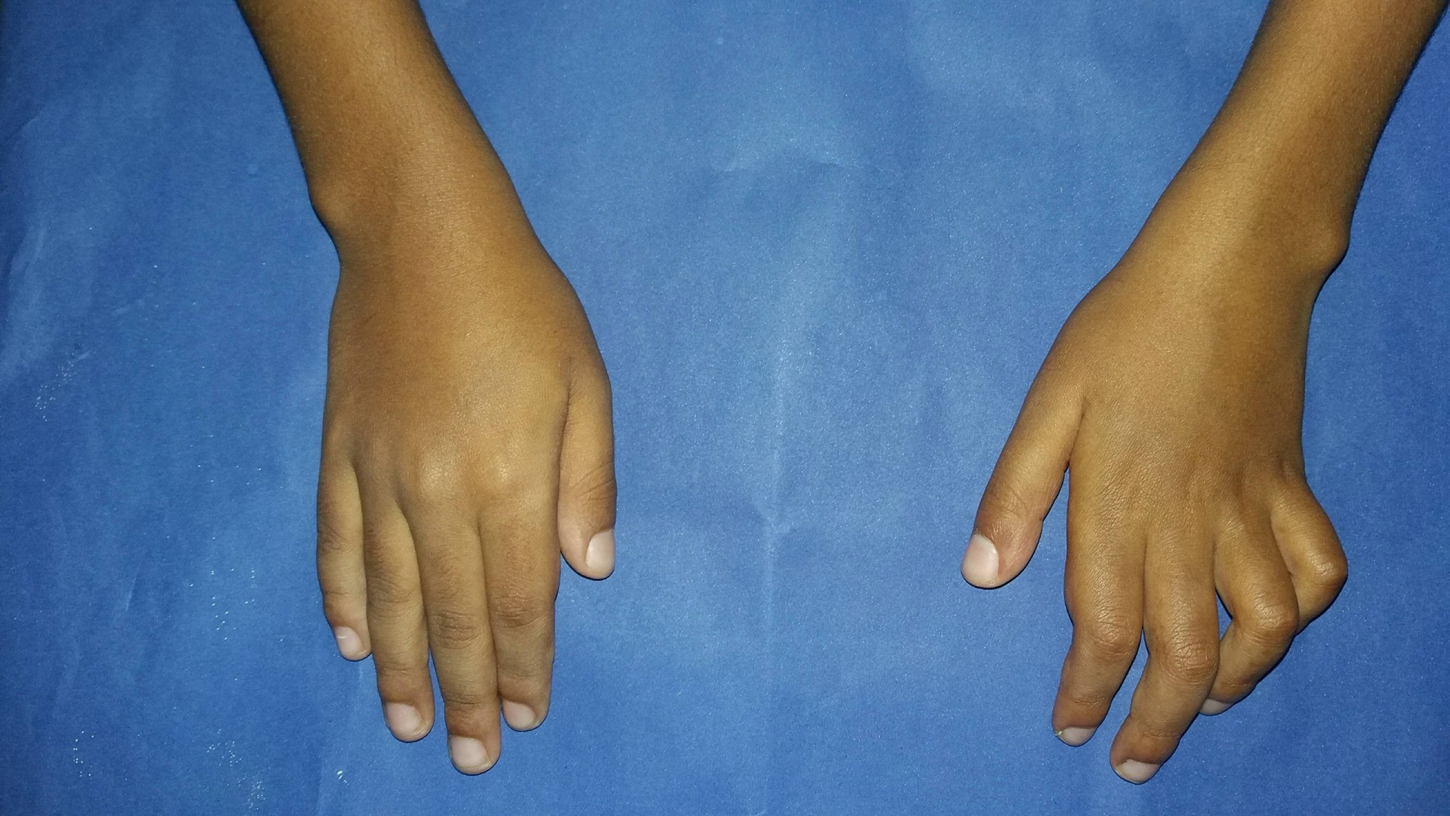
Median-cubital claw deformity in the left hand: cubital and median nerves affected at diagnosis. Nine-year-old child with borderline leprosy.

**Fig 5:**
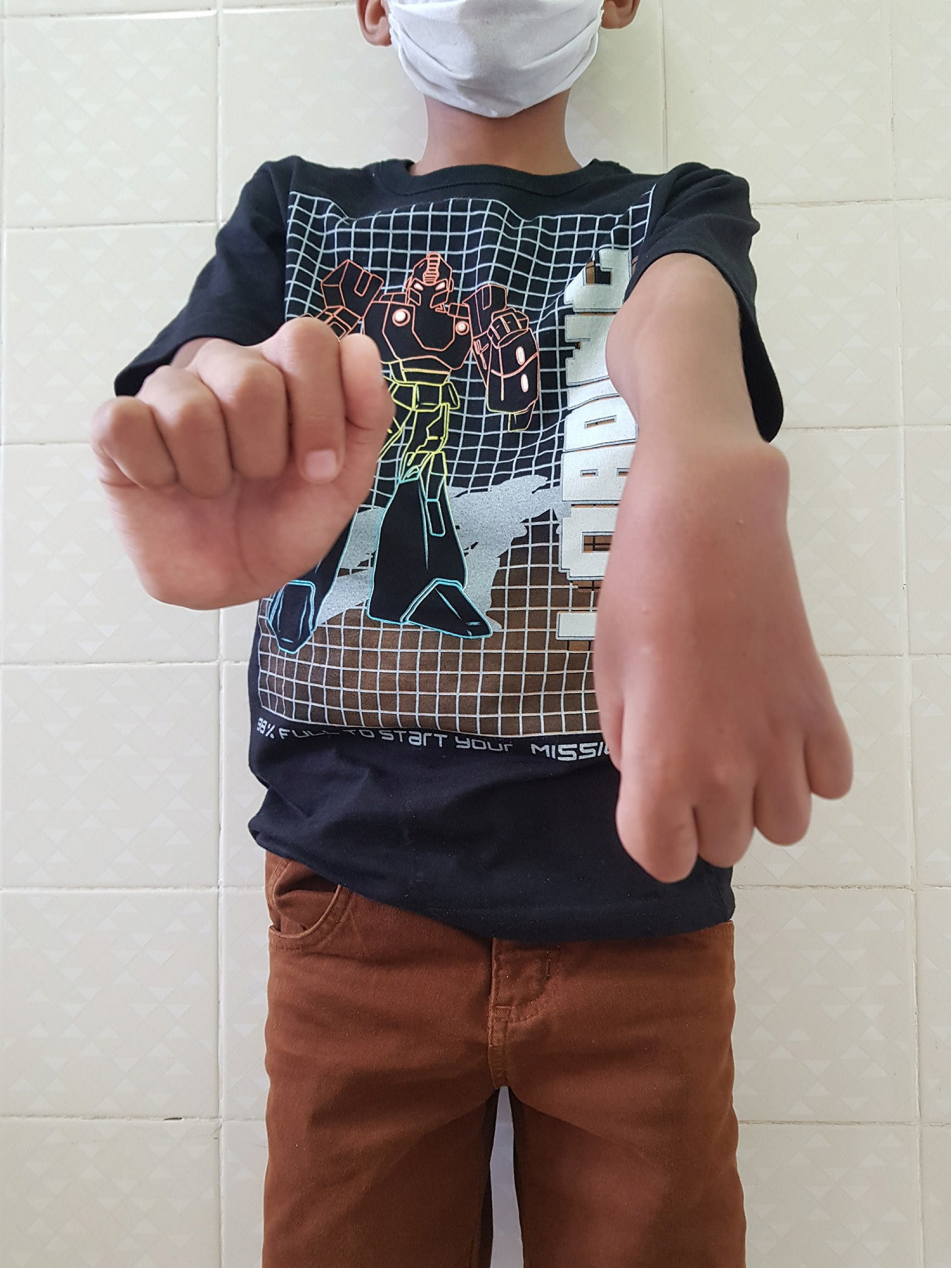
Dropped hand deformity: worsening of neural damage, with damage radial, cubital and median nerves trunks. From the same nine-year-old child with borderline leprosy.

According to the progression of leprosy reaction; 57.9% (22/38) presented the condition of non-progression, that is, 34.2% (13/38) unchanged the neural picture of the diagnosis and 23.7% (9/38) improved their initial neurological impairment. However 42.1% (16/38) patients had progression of the leprosy reaction with worsening of the initial nerve damage (Figs 4 and 5). On analyzing the association between the progression of the leprosy reaction and clinical and epidemiologic characteristics, the following variables showed significant associations (p≤0.05): number of reaction episodes (two or more), nerve damage at diagnosis, number affected nerves at diagnosis (two or more), isolated neuritis, silent neuritis and low educational level of caregiver (incomplete elementary school) (Table 3).

**Table 3:** Association between the progression of the leprosy reaction and clinical, epidemiologic, reactions and nerve damage characteristics.

Variables (n = 38)	Non-progression (n = 22), n (%)	Progression (n = 16), n (%)	*P* *
**Number of reaction episodes**			
Only one	**9 (40.9)**	**1 (6.3)**	**0,0250**
2 or more	**13 (59.1)**	**15 (93.7)**	
**Nerve damage at diagnosis**			
Yes	**10 (45.5)**	**13 (81.3)**	**0,0435**
No	**12 (54.5)**	**3 (18.7)**	
**Number of nerves affected at diagnosis**			
0–1	**19 (86.4)**	**7 (43.7)**	**0,0116**
2 or more	**3 (13.6)**	**9 (56.3)**	
**Reaction types**			
Isolated neuritis	**5 (22.7)**	**11 (68.8)**	**0,0077**
Reaction with skin lesions	**17 (77.3)**	**5 (31.2)**	
**Silent neuritis**			
Yes	**6 (27.3)**	**10 (62.5)**	**0,0471**
No	**16 (72.7)**	**6 (37.5)**	
**Educational level of caregivers**			
Incomplete elementary school	**8 (36.4)**	**13 (81.3)**	**0,0088**
≥ Complete elementary school	**14 (63.6)**	**3 (18.7)**	

*Fisher exact test.

## Discussion

This was an important study because it is the first where children with leprosy reaction were followed up during MDT treatment and in the post-discharge period, describing how these complications evolve in childhood and which factors are associated with the worst clinical prognosis.

A high proportion (42.1%) of the children followed evolved with progression of the leprosy reaction, that is onset or worsening of the peripheral nerve damage and only 23.7% improved. These findings suggest that the children in the study had old nerve damage, idea reinforced by the fact that most have already been diagnosed with peripheral nerve damage and some degree of physical disability. The management and control of the inflammatory process in the nerve seems to be a great challenge. In a randomised study of treatment for leprosy reactions in India, showed a poor improvement rate of nerve damage, particularly if there was old damage [18]. It may seem ambiguous to speak of old impairment nerve in children, however in the Brazilian Amazon, underdiagnosing of leprosy and difficulty and delay in diagnosis among children, are frequent situations [5,11]. Early diagnosis of leprosy in adittion early diagnosis of reactions and neuritis, with appropriate immediate treatment, can help prevent the onset and/or worsening of physical disabilities and deformities still at pediatric age.

Two or more reaction episodes, nerve damage at diagnosis, multiple nerves affected at diagnosis, isolated neuritis, silent neuritis and low educational level of caregiver were variables related to reaction progression in the study children.

The association between leprosy reactions and neural damage resulting in disabilities is widely documented in the literature [19,1,20,21]. Reactions increase the risk of worsening the degree of physical disability. In a study carried out in a hyperendemic municipality in the state of Mato Grosso, Brazil, the occurrence of reactional episodes was one of the main risk factors for worsening the physical disability of patients in the post-discharge period [22]. Half of the children followed in this study had reactional episodes in the period after MDT discharge which justifies ongoing monitoring. It is necessary to guarantee health care with systematic monitoring, especially of the patient’s neurological condition, including in the post-discharge period [23,22].

It is worth noting that the vast majority of the evaluated children had recurrent reactional episodes. The concept of a leprosy reaction as a single isolated episode and that new episodes rarely occur [16], has already become obsolete. Studies with adult leprosy patients demonstrated that recurrence of reactional episodes, especially type 1, is frequent, ranging from 30 to 50% [24,18]. Furthermore, it is also known that it is difficult to identify whether a subsequent reaction event is a new episode or only a continuation of the first [25]. Another possible explanation for the high recurrence of reactions in the children in the study is that it was not investigated co-infections. Oral co-infections, for example, are very common in children in situations of social vulnerability, can be a pro-inflammatory factor causing refractoriness of reactional episodes [26].

The variable two or more affected nerves at diagnosis was also associated with worse clinical prognosis of the leprosy reaction in line with previous studies in various age groups of leprosy patients [19,27,28,20]. Marjority of the children participating in the study had nerve damage. It is the damage to the peripheral nerves that causes the disabilities and deformities associated with leprosy [29]. In this way, it is necessary to remove children with severe clinical forms, reactions and disabilities from invisibility. Especially in highly endemic areas, in addition to early diagnosis of the disease, it is necessary to think about children who have already been diagnosed with severe forms and present complications, adapting instruments for assessing neurological impairment and pain in addition to adapting rehabilitation and self-care techniques to the range pediatric age.

Silent neuritis and isolated neuritis are other variables associated with a worse reactional prognosis. A possible explanation for this fact would be that when the warning signs and symptoms of the reactional episode are absent, as in the case of isolated neuritis (without exacerbation of skin lesions) and/or silent neuritis (absence of pain), this makes it difficult for the child, the caregiver and even the health team realizes that there is a worsening of the neurological condition and consequently the need for intervention by the health team. In a study conducted in China, less than 2% of the 5000 symptoms analyzed in 2125 patients newly diagnosed with leprosy reported pain, in addition, nervous symptoms were associated with a longer time to diagnosis when compared with cutaneous and cutaneous and nervous symptoms [21].

Silent neuritis occurred in more than forty percent followed up children. There are few studies investigating silent neuritis and it’s incidence in leprosy patients in general is around 5% [30,31]. However, in an old study on the treatment of leprosy reactions, one third of all patients had silent neuritis [32] and in another research with a prospective cohort performed in Bangladesh, 86% of leprosy patients that developed nerve damage after diagnosis it was silently, that is, without a skin reaction or pain [25]. Another hypothesis is that the painful experience in children may be different from adults [33]. Pain in childhood is difficult to assess and in children with leprosy it is still an unexplored subject in need of studies.

The study revealed that low educational level of caregiver (incomplete elementary school) was associated with the worsening neurological impairment of the children. The little access to education may make it difficult for the caregiver to perceive at an early stage of leprosy reaction and/or hinder the understanding and execution of rehabilitation techniques in children, favoring of the negative evolution of this complication. Previous studies had already shown that in leprosy patients adults the low level education directly influences the poor understanding about disease and their preventive, therapeutic measures and self care techniques, favoring ocurrence of physical disabilities [28,34]. Another hypothesis is that also the lack of knowledge about reaction symptoms and the possibility of nerve damage with the evolution of physical disabilities can also be an aggravating factor, after all, in another study with 65 children and adolescents with leprosy in India, only 26.2% knew that leprosy could lead to physical disabilities [35]. Therefore, it is necessary to break the academic paradigm that leprosy is an adult disease due to its long incubation period and when it affects children is in mild, paucibacillary form. In highly endemic regions, the disease in childhood often occurs in severe forms and complications such as reactions and neural damage are not uncommon.

In conclusion, we observed that children are even diagnosed with old neural damage, presenting recurrent reactional episodes evolving with worsening neurological impairment. Reactions can also occur silently, that is, without pain and only in the nerve, without exacerbation of skin lesions, therefore, systematic monitoring, with an emphasis on neurological impairment, including in the post-discharge period from MDT, is of fundamental importance to minimize the emergence or worsening of disabilities. Furthermore, the education of family members and their engagement in perceiving reactional episodes and helping the child to carry out rehabilitation and self-care is a key part of a good prognosis.

As a limitation of this study there is the fact that the children monitored were diagnosed in a leprosy reference service, which therefore tends to have more severe cases and complications than in the general community.

## Supporting information

S1 File Values of sensory and motor functions of patients in the initial and final evaluation, emergence of deformities and its development. (PDF)
