# Supplementary material for: Progression of the leprosy reaction and nerve damage: A prospective cohort study in children with leprosy from the Brazilian Amazon
Source: PLoS Negl Trop Dis. 2024 Dec 19;18(12):e0012772. doi: 10.1371/journal.pntd.0012772 (PMC11698567; doi:10.1371/journal.pntd.0012772)
Supplement: S1 File — (PDF) [file pntd.0012772.s001.pdf]

Support Information

Values of sensory and motor functions of patients in the initial and final evaluation, emergence of deformities and its development

Patient	Initial strength	Initial sensitivity	Final strength	Final sensitivity	Appearance new deformities	Initial total	Final total	Development
01	42	152	46	169	no	194	215	improved
02	50	200	44	191	no	250	235	worsened
03	50	183	50	164	no	233	214	worsened
04	49	203	48	201	no	252	249	worsened
05	50	201	50	204	no	251	254	improved
06	50	216	50	216	no	266	266	unchanged
07	47	210	48	202	no	257	250	worsened
08	50	203	50	211	no	253	261	improved
09	50	215	50	205	no	265	255	worsened
10	50	216	50	216	no	266	266	unchanged
11	46	215	47	210	no	261	257	worsened
12	50	216	50	216	no	266	266	unchanged
13	50	216	50	216	no	266	266	unchanged
14	43	79	47	146	bone resorption	122	193	worsened
15	50	216	48	207	no	266	255	worsened
16	48	215	50	216	no	263	266	improved
17	50	216	50	216	no	266	266	unchanged
18	49	216	50	216	no	265	266	improved
19	47	201	48	206	no	248	254	improved
20	50	205	50	213	no	255	263	improved
21	50	216	50	216	no	266	266	unchanged
22	50	216	50	216	no	266	266	unchanged
23	50	216	48	209	no	266	257	worsened
24	43	148	39	122	bone resorption	191	161	worsened
25	50	213	47	185	fallen hand	263	232	worsened
26	49	207	46	201	no	256	247	worsened
27	50	216	50	216	no	266	266	unchanged
28	50	216	50	216	no	266	266	unchanged
29	50	216	50	216	no	266	266	unchanged
30	50	216	50	216	no	266	266	unchanged
31	50	216	50	216	no	266	266	unchanged
32	45	202	44	176	no	247	220	worsened
33	47	163	47	174	no	210	221	improved
34	47	206	48	208	no	253	256	improved
35	48	213	46	197	no	261	243	worsened
36	50	216	50	216	no	266	266	unchanged
37	50	175	50	173	no	225	223	worsened
38	49	194	48	202	bone resorption	243	250	worsened
